# Oxygen Sparing Effect of Bacteriotherapy in COVID-19

**DOI:** 10.3390/nu13082898

**Published:** 2021-08-23

**Authors:** Giancarlo Ceccarelli, Massimiliano Marazzato, Luigi Celani, Francesca Lombardi, Alessandra Piccirilli, Massimo Mancone, Vito Trinchieri, Francesco Pugliese, Claudio M. Mastroianni, Gabriella d’Ettorre

**Affiliations:** 1 Department of Public Health and Infectious Diseases Sapienza, University of Rome, 00185 Rome, Italy; giancarlo.ceccarelli@uniroma1.it (G.C.); luigi.celani@uniroma1.it (L.C.); vito.trinchieri@uniroma1.it (V.T.); claudiomaria.mastroianni@uniroma1.it (C.M.M.); gabriella.dettorre@uniroma1.it (G.d.); 2 Department of Life, Health & Environmental Sciences, University of L’Aquila, 67100 L’Aquila, Italy; francesca.lombardi@univaq.it; 3 Department of Biotechnological and Applied Clinical Sciences, University of L’Aquila, 67100 L’Aquila, Italy; alessandra.piccirilli@univaq.it; 4 Department of Clinical Internal Anesthesiologic and Cardiovascular Sciences Sapienza, University of Rome, 00185 Rome, Italy; massimo.mancone@uniroma1.it; 5 Department of Anesthesia and Intensive Care Medicine, Sapienza University of Rome, 00185 Rome, Italy; francesco.pugliese@uniroma1.it

**Keywords:** COVID-19, SLAB51, probiotics, nitric oxide, hypoxia

## Abstract

Background: We previously reported that severe COVID-19 patients had higher chances of survival and a reduced risk of developing respiratory failure when administered with the probiotic formulation SLAB51. This study aimed to investigate further bacteriotherapy mechanisms and how early they are activated. Methods: We performed an analysis on the blood oxygenation parameters collected in sixty-nine severe COVID-19 patients requiring non-invasive oxygen therapy and presenting a CT lung involvement ≥50%. Twenty-nine patients received low-molecular-weight heparin, azithromycin and Remdesivir. In addition, forty subjects received SLAB51. Blood gas analyses were performed before the beginning of treatments and at 24 h. Results: The patients receiving only standard therapy needed significantly increased oxygen amounts during the 24 h observation period. Furthermore, they presented lower blood levels of pO_2_, O_2_Hb and SaO_2_ than the group also supplemented with oral bacteriotherapy. In vitro data suggest that SLAB51 can reduce nitric oxide synthesis in intestinal cells. Conclusions: SARS-CoV-2 infected patients may present lesions in the lungs compromising their gas exchange capability. The functionality of the organs essential for these patients’ survival depends mainly on the levels of pO_2_, O_2_Hb and SaO_2_. SLAB51 contains enzymes that could reduce oxygen consumption in the intestine, making it available for the other organs.

## 1. Introduction

The role of oral bacteriotherapy in the armamentarium for fighting the COVID-19 pandemic is under scrutiny [1,2,3]. In a previous retrospective study of 200 patients, we showed that subjects treated with a specific formulation of bacteria (SLAB51) had a significantly higher chance of survival [4]. Moreover, we reported that the risk of developing respiratory failure for a patient is reduced eightfold by administering SLAB51 in addition to the routinely used therapy (RUT) [5].

The present work aimed to investigate further SLAB51’s short-term action in alleviating the respiratory conditions in subjects presenting SARS-CoV-2 infections. To this end, we examined the response of two groups of patients, one treated with RUT and one with RUT and bacteriotherapy twenty-four hours after the product’s initial administration. We offer evidence that SLAB51 contains bacteria endowed with arginine deiminase activity (ADI), which catalyzes L-arginine’s hydrolysis to citrulline and ammonia. ADI inhibits the arginine-dependent synthesis of nitric oxide (NO). This gas is a signaling molecule for the innate inflammatory immune response and modulates intestinal vasodilation. Furthermore, ADI, by depleting arginine, may exert antiviral activity against some viruses, including SARS-CoV-2, and decrease the hyperinflammatory lung damage characteristics of patients with severe COVID-19 [6,7].

## 2. Materials and Methods

### 2.1. Design of the Study, Population, Settings and Data Collection

This study is a “real life” prospective analysis of adult (>18 years) SARS-CoV-2 infected patients supported with oxygen therapy progressively hospitalized between October 2020 and December 2020 in the Policlinico Umberto I, University “Sapienza”, Rome, Italy.

**Figure 1:**
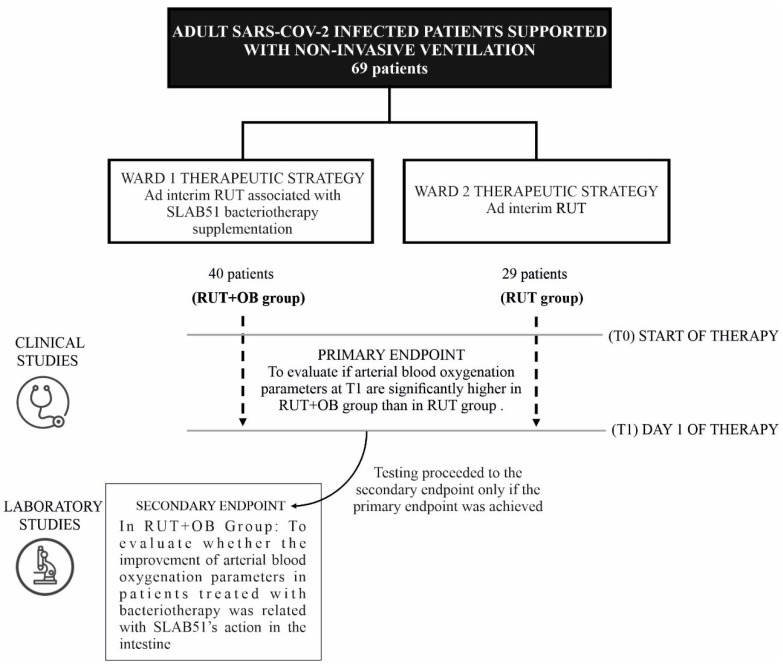
Schematic representation of the study design.

The patients included in the study were hosted in two different wards devoted to the management of COVID-19: in the first ward, a clinical therapeutic strategy including ad interim routinely used therapy associated with SLAB51 bacteriotherapy supplementation was adopted (RUT+OB group), while in the second ward, only RUT was administered (Figure 1). The two wards had similar characteristics regarding the type of hospitalized patients and the expertise of the healthcare personnel. Inclusion criteria were patients >18 years old, a diagnosis of COVID-19, a need of oxygen support and a CT scan confirming diagnosis of SARS-CoV-2 related pneumonia. Exclusion criteria were pregnancy, other previous chronic CT chest diseases such as pulmonary edema and interstitial lung disease. All patients clinically diagnosed with COVID-19 underwent a high-resolution CT/non-contrast enhanced chest CT at admission in the hospital. Two multidetector CT scanners (Somatom Sensation 16 and Somatom Sensation 64; Siemens Healthineers) were used for all examinations. Radiologists, experts in thoracic imaging, performed the CT image analysis assessing location and distribution of the disease and CT chest findings. A semi-quantitative CT score was calculated based per each of the 5 lobes considering the extent of anatomic involvement, as follows: 0, no involvement; 1, <5% involvement; 2, 5–25% involvement; 3, 26–50% involvement; 4, 51–75% involvement; and 5, >75% involvement. The resulting global CT score was the sum of each individual lobar score (0 to 25). The sources for patient data were medical records stored in the electronic information system of the wards involved. The variables considered included: (1) anamnestic data, (2) past clinical history (comorbidities) and (3) current clinical history, treatment and laboratory data. The study’s primary endpoint was the evaluation of differences in partial pressure of oxygen (pO_2_), oxygenated hemoglobin (O_2_Hb) and the percentage of hemoglobin saturated with oxygen (SaO_2_) at baseline (T0) and after 24 h from the start of therapy (T1) in the two groups to confirm/refute previous observations [5]. The arterial blood gas (ABG) test was performed using the blood drawn from the radial artery while the fraction of inspired oxygen (FiO_2_) as well as the amount of supplied oxygen (L/min) were also registered. In case of achievement of the primary outcome, the secondary endpoint was to evaluate whether the improvement of arterial blood oxygenation parameters in patients treated with bacteriotherapy was due to SLAB51’s action in the intestine. In some patients, a blood sample was obtained after 5–6 h from the bacteriotherapy administration to evaluate how early the phenomenon was occurring.

### 2.2. Diagnosis of SARS-CoV-2 Infection

The diagnosis of SARS-CoV-2 infection was defined as one positive oropharyngeal and nasopharyngeal swab performed in duplicate for SARS-CoV-2 E and S gene by a reverse transcriptase-polymerase chain reaction (RT-PCR).

### 2.3. COVID-19 Treatments

The patients were treated with ad interim RUT as suggested by the provisional guidelines of the Italian Society of Infectious and Tropical Diseases (SIMIT) and the Italian Medicine Agency (AIFA). In detail: Dexamethasone (6 mg daily for 10 days) plus low molecular weight heparins (prophylactic dosage) ± azithromycin (500 mg daily); Remdesevir, as per AIFA guidelines. Bacteriotherapy (SLAB51) was administered in three equal doses per day for a total of 2400 billion bacteria per day. SLAB51 contains *Streptococcus thermophilus* DSM 32245, *Bifidobacterium lactis* DSM32246, *Bifidobacterium lactis* DSM 32247, *Lactobacillus acidophilus* DSM 32241, *Lactobacillus helveticus* DSM 32242, *Lactobacillus paracasei* DSM 32243, *Lactobacillus plantarum* DSM 32244 and *Lactobacillus brevis* DSM 27961 (Sivomixx800^®^, Ormendes SA, Lausanne, Switzerland).

### 2.4. Supplemental Oxygen

All patients included in the study were supported by oxygen therapy delivered via Venturi masks in spontaneous breathing patients.

### 2.5. Ethics Committee Approval

Reporting of the study conforms to broad EQUATOR guidelines. The Ethics Committee of Policlinico Umberto I approved the study with number 109/2020.

### 2.6. Arginine Deiminase (ADI) Assay

SLAB51 was suspended at the concentration of 133 × 10^9^ CFU in 10 mL of phosphate buffered saline (PBS, EuroClone, West York, UK). ADI activity was analyzed by the amount of citrulline formed from arginine by measuring colour changes due to the products formed by diacetyl-mono-oxime as previously described [8].

### 2.7. Cell Line, Culture Condition and Treatment

Human colon adenocarcinoma-derived CaCo-2 cell lines purchased from Sigma-Aldrich (St. Louis, MO, USA) were maintained in culture in DMEM supplemented with 10% FCS, 1% non-essential aminoacid, 1 mM sodium pyruvate and 2 mM L-glutamine, 100 U/mL penicillin and 100 μg/mL streptomycin (EuroClone, West York, UK). After reaching 80% confluence cells were seeded into a sterile tissue culture 12-well plate (Becton Dickinson, San Jose, CA, USA) at 40,000 cells/cm^2^ and allowed to attach overnight. The cells were then treated for 24 h with or without nitric oxide synthase 2 (NOS2) inhibitor N-(3-(aminomethyl) benzyl) acetamidine (1400 W) (Sigma-Aldrich, Saint Louis, MO, USA) (100 µM), ADI inhibitor formamidine hydrochloride (Sigma-Aldrich) (10 mM) or SLAB51 (10^7^ CFU/mL, suspended at the concentration of 133 × 10^9^ CFU in 10 mL of phosphate buffered saline (PBS) (EuroClone, West York, UK). Where specified, a SLAB51 sample was preincubated for 30 min with or without formamidine (100 mM). No significant influence on the cell viability compared to the control cells was registered after the treatment with SLAB51, 1400 W and formamidine, as evaluated by Trypan blue assay.

### 2.8. Total RNA Extraction and NOS2 Expression by RT-PCR

Total RNA from CaCo-2 cells was extracted using TRIzol reagent according to the manufacturer’s protocol. RNA for NOS2 positive control was obtained from murine macrophage RAW 264.7 cells (Sigma-Aldrich) treated with IFN-γ 100 ng/mL and LPS 1 µg/mL. Negative control was represented by acute human T leukemia-derived Jurkat cells (ATCC). RT-PCR was performed as previously described [9]. All PCR reagents and EuroSafe Nucleic Acid Staining Solution were acquired from EuroClone. Primers were acquired from IDT (Integrated DNA Technologies, Coralville, IA, USA). The RT-PCR reaction was made by the Thermocycler GeneAmp PCR System 9700 (Applied Biosystems, Foster City, CA, USA). PCR quantification was performed by densitometer (UVItec Limited BTS −20 M, Cambridge, UK) and the densitometric analysis was made using ImageJ software. The primers used for NOS2 and beta-actin amplification were as follows: NOS2 FOR5′-CTGACGGGAGATGAGCTC- 3′; NOS2 REV5′-AGTCGTGCTTGCCATCACTC- 3′; β-actin FOR5′-AGCGGGAAATCGTGCGTG-3′; β-actin REV5′-CAGGGTACATGGTGGTGCC-3′.

### 2.9. Nitrite Level Assay

The enzymatic activity of NOS2 was evaluated by measuring nitrite levels using nitrate reductase and the Griess reaction through a colorimetric assay (Nitrite Assay kit, Sigma-Aldrich Co., Milan, Italy) in the supernatants of CaCo-2 cells, treated as described, according to the manufacturer’s instructions.

### 2.10. Statistical Analysis

No sample size calculations were performed. The categorical variables were compared using the χ2 test with Yates’ continuity correction and showed as absolute frequencies and percentage. The bilateral Mann–Whitney U test was used for continuous variables to determine statistically significant differences between groups at each considered time points, while for each group the Wilcoxon test was used to assess significant differences between consecutive time points. For nitrite levels, the non-parametric Kruskal–Wallis test followed by Dunn’s post hoc test were used to evaluate statistical significance among tested conditions. In each case, a *p* value ≤ 0.05 was considered statistically significant. Analyses were performed by using the statistical software R 4.0.3 [10].

## 3. Results

### 3.1. Primary Endpoint

Data were collected and compared between COVID-19 positive patients who received RUT plus oxygen (29; 42%) and subjects who were also treated with oral bacteriotherapy (40; 58%) plus oxygen. The main characteristics of both groups of patients are summarized in Table 1.

**Figure 2:**
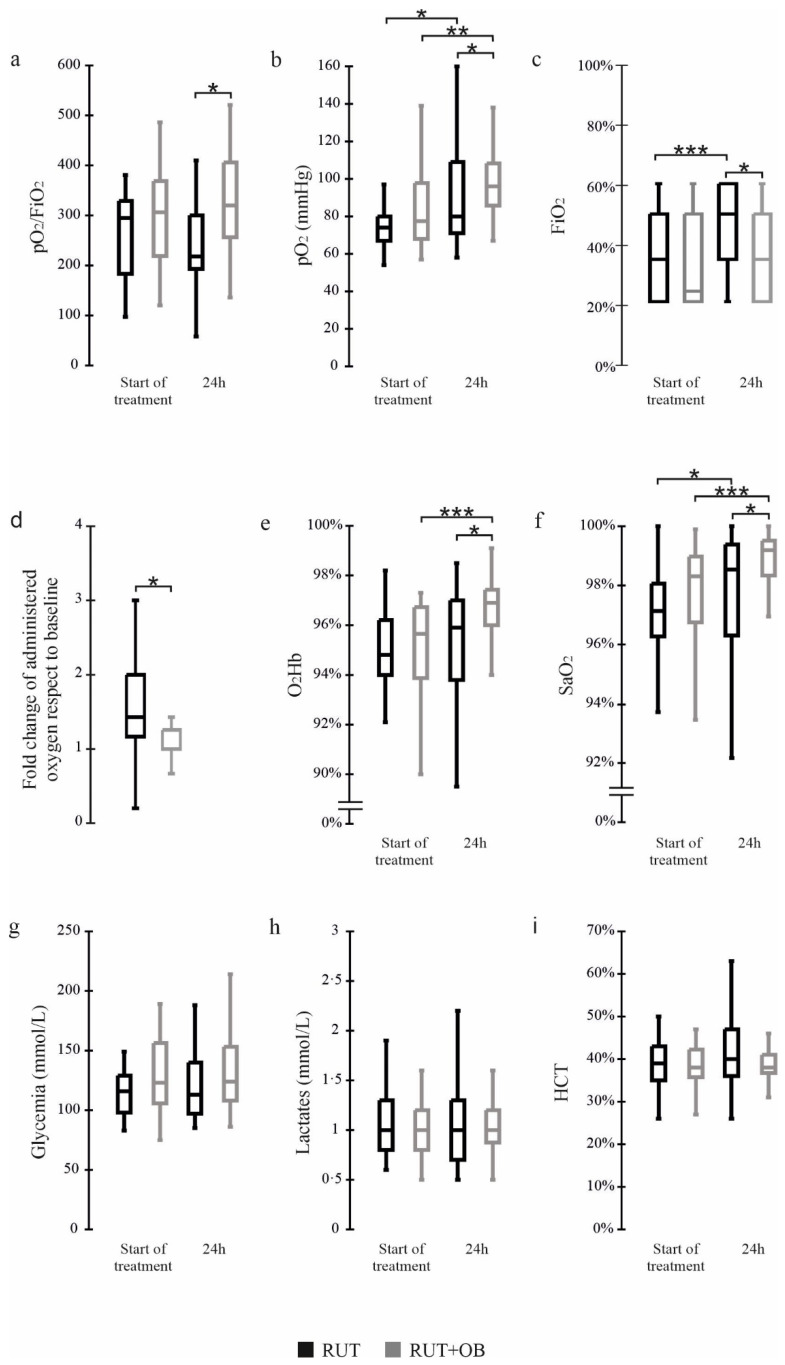
Results of blood gas analyses. Box end whiskers plots showing the value distribution of (**a**) PO_2_/FiO_2_ ration, (**b**) pO_2_, (**c**) FiO_2_, (**d**) Fold change of administered oxygen respect to baseline, (**e**) O_2_Hb, (**f**) SaO_2_, (**g**) glycemia, (**h**) lactates, and (**i**) HTC obtained by routine laboratory analysis. Where present, statistical significance has been reported between RUT and RUT+OB groups at each time point, as well as for each group over time. *: *p* ≤ 0.05; **: *p* ≤ 0.001; ***: *p* ≤ 0.0001.

Except for sex, at baseline, the two groups determined on the base of SLAB51 administration were homogeneous for all the considered variables comprising the SARS-CoV-2 drug therapies, the pO_2_ levels, the pO_2_/FiO_2_ ratio and the amount of administered oxygen (median; IQR RUT 4; 1–6 L/min; RUT+OB 1.5; 1–6 L/min, *p* = 0.31). After 24 h from the first dose of SLAB51, the RUT+OB group showed significantly higher values of the pO_2_/FiO_2_ ratio (*p* = 0.002) and pO_2_ (*p* = 0.01) than the RUT group, evidencing improved conditions (Figure 2a,b). Concerning the FiO_2_ values, at the beginning of the observation period the two groups were homogeneous while, at the 24 h endpoint, significantly higher FiO_2_ levels were registered for the RUT group than the RUT+OB one (*p* = 0.002) (Figure 2c). An analysis of O_2_Hb and SaO_2_ levels yielded results in line with those previously described for the pO_2_ and pO_2_/FiO_2_ ratio with significantly higher values registered for the RUT+OB group respective to the group receiving only standard therapy (O_2_Hb, *p* = 0.006; SaO_2_, *p* = 0.038) (Figure 2e,f). Notably, after 24 h from the beginning of treatment, the group administered with SLAB51 presented better blood oxygenation levels than the group receiving only the standard therapy, despite the fact that the RUT group registered a significantly higher increase in the amount of supplied oxygen over time (*p* = 0.002) (Figure 2d). Finally, the two groups were homogeneous for glycemia, lactates and hematocrit levels both at the start of treatment and at the following 24 h (Figure 2g–i).

We were also able to obtain data relative to 6 h from the start of treatments in a few subjects treated with RUT or RUT+OB. The pO_2_/FiO_2_ ratio, pO_2_, SaO_2_ and O_2_Hb improved in the RUT+OB group and were unchanged or worsened in the RUT group. (Table 2).

The hospital registry allowed us to see how the disease progressed in patients treated with RUT or RUT + OB during their hospitalization over the following weeks. Four (13.8%) patients treated with RUT were transferred to the ICU due to worsening respiratory conditions. In addition, one of these patients died. Only one (2.5%) patient treated with RUT and oral bacteriotherapy developed a clinical picture that necessitated transfer to the ICU, and no subject in this group died.

### 3.2. Secondary Endpoint

**Figure 3:**
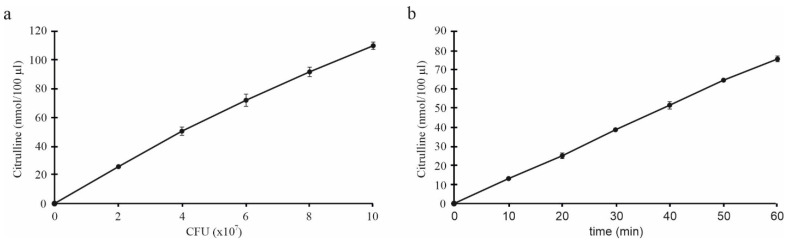
ADI activity in SLAB51 sample. (**a**) Citrulline levels (nmol/100 µL) after 1 h incubation with different amounts of SLAB51 suspension (×10^7^/100 µL); (**b**) kinetics of ADI activity of SLAB51 at 6 × 10^7^ CFU/100 µL. Data are expressed as mean of two measurements ± SD.

The enzymatic activity of ADI expressed as nmol/100 µL citrulline levels was first analyzed at different concentrations of the SLAB51 sample at one-hour incubation. The results showed that the ADI activity was proportional to the SLAB51 amount, as shown in Figure 3a. The test was repeated with a fixed concentration of bacterial suspension (6 × 10^7^ CFU/100 µL) and evaluated at 10-min intervals, thus highlighting that the ADI activity of whole bacteria was stable and linear over assay time; of interest, the capacity of the enzyme to convert arginine in citrulline was detectable as early as a few minutes (Figure 3b).

**Figure 4:**
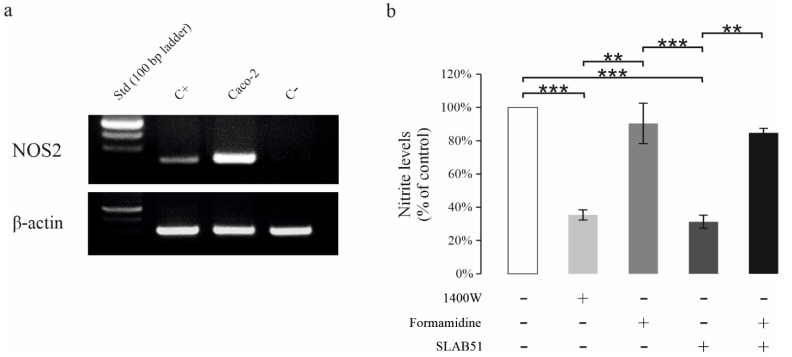
Effect of SLAB51 on NO production in CaCo-2 cells. CaCo-2 cells basically expressed high levels of NOS2 as shown in the representative RT-PCR (**a**). C+: positive control (RAW cells stimulated with IFN-γ + LPS); C- = negative control (Jurkat cells). Nitrite levels were determined in the supernatants from CaCo-2 cells treated for 24 h (**b**) with or without NOS2 inhibitor 1400 W (100 µM), ADI inhibitor formamidine (10 mM) or SLAB51 (10^7^ CFU) preincubated for 30 min with or without formamidine. Values are expressed as percentage of nitrite levels vs. control (mean ± SD). For comparative analysis of data groups, the Kruskal–Wallis test followed by Dunn’s post hoc test was used (** *p* < 0.01, *** *p* < 0.001).

We next tested the ability of SLAB51 to inhibit NOS2 activity, using the human colon adenocarcinoma-derived CaCo-2 cells, which constitutively express high levels of NOS2 as shown by the RT-PCR analysis (Figure 4a), thus confirming previous reports [11]. The NO release in CaCo-2 cells after 24 h was measured as nitrites levels (µM) in culture medium and expressed as a percentage of nitrite levels vs. control (Figure 4b). Exposure to 1400 W, one of the most potent and selective NOS2 inhibitors was used to control NOS2 inhibition in our experimental model positively. As expected, 1400 W significantly reduced nitrite levels [12]. SLAB51 treatment reduced NO release to a level comparable to that of 1400 W at 24 h. To investigate the potential involvement of ADI in the ability of the SLAB51 to inhibit NOS2 activity, formamidine, a specific inhibitor of ADI was used [13]. The preincubation of SLAB51 with formamidine completely abrogated the ability of SLAB51 to inhibit NO production, bringing back the nitrite value to the control level. Cell exposure to formamidine alone did not affect nitrite levels, excluding the fact that formamidine could directly influence NOS2 activity.

## 4. Discussion

**Figure 5:**
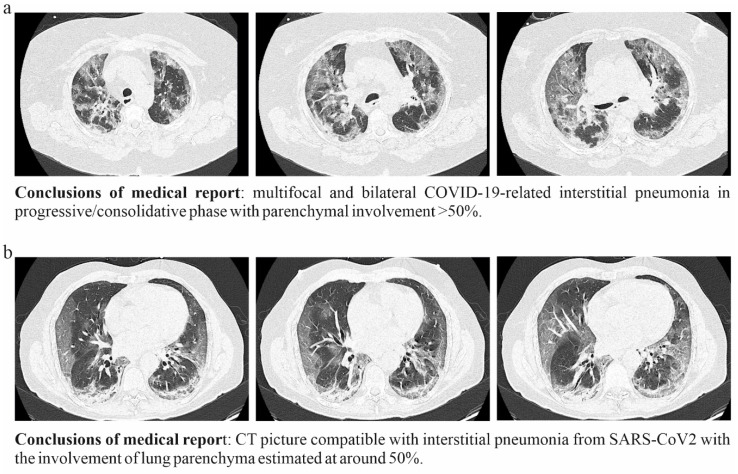
CT lung scan pictures of two randomly chosen COVID-19 patients (**a**,**b**) enrolled in the study.

The COVID-19 disease is characterized by extremely variable outcomes ranging from the complete absence of symptoms to severe pneumonia and acute respiratory distress syndrome (ARDS) [14]. Under these clinical conditions, mechanical ventilation might become a needed choice. The objective is to give the body as much oxygen as possible, so the brain, heart and kidneys maintain efficiency while waiting for the lungs to heal. Unfortunately, prolonged mechanical ventilation can exacerbate lung dysfunction. Overdistention from high tidal volumes increases airway pressure, or inadequate positive end-expiratory pressure may cause ventilator-induced lung injury. The damage of the alveolar-capillary barrier activates mediators and inflammatory cells that may further increase the biotrauma of COVID-19 patients, especially if they are elderly and with pre-existing organ diseases. Therefore, it is clear that every opportunity to avoid mechanical ventilation is to be considered [15]. Our group supports the use of oral bacteriotherapy as adjuvant therapy for the management of COVID-19 subjects [4,5]. The data presented in this paper confirm its utility in two groups of patients initially comparable in terms of clinical, respiratory and laboratory parameters. After 24 h, from the start of bacteriotherapy, the group treated with SLAB51 presented improved blood oxygenation compared to the group receiving only RUT, as evidenced by the analysis of pO_2_, O_2_Hb and SaO_2_ values. This improvement in oxygenation parameter coupled with the feeling of well-being reported by several patients supplemented with SLAB51. Notably, although the group treated with only RUT was supplied with significantly increased amounts of oxygen during the 24 h observation period, at the endpoint, it presented substantially lower blood oxygenation than that observed for the RUT+OB one. According to surface tension and La Place’s law, during ARDS and pneumonia, the alveoli tend to become flooded entirely or not flooded at all. External oxygen furniture to increase the O_2_ quantity provided to the patient may not guarantee the hypoxemia resolution. The amount of absorbed oxygen is limited mainly by the finite gas exchange capability of damaged alveoli, combined with the reduced number of alveolar cells effectively working in COVID-19 compromised lungs. Consequently, the significantly lower pO_2_/FiO_2_ ratio registered values, at 24 h, for the RUT group confirms the tendency to a worsening of hypoxemia when only the standard therapy is administered. Previously published studies suggest that the recovery of lung injury associated with COVID-19 is slow and that, in severely ill patients, pulmonary troubles can persist for a long time after hospital discharge [16,17]. The subjects enrolled in our study had a CT lung involvement ≥ 50%. Figure 5 displays the exemplificative CT scan pictures relative to two patients randomly chosen within the whole analyzed population. It is common clinical knowledge that an effective lung recovery certainly takes more than hours in these conditions. The improvement of the oxygenation parameters within a few hours from the administration of SLAB51 suggests that this product modulates the blood consumption of oxygen in the gut. Less oxygen consumption by the intestinal cells results in more oxygen available for other organs.

**Figure 6:**
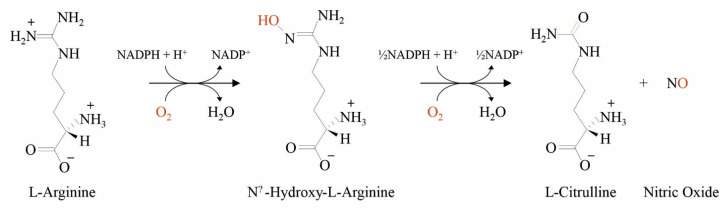
Biochemical reaction describing the catalytic activity of NOS in NO formation. The reaction consumes 1·5 mol of NADPH and 2 mol of oxygen.

The estimated surface area of the gastrointestinal tract is around 250–300 m^2^, and under normal conditions, the intestinal mucosa receives between 10% and 35% of the total cardiac output [18]. Our in vitro assays evidenced that the bacteria in SLAB51 are characterized by high ADI activity and are associated with a significant short-time reduction in the NO produced by cells mimicking the intestinal epithelia. ADI catalyzes L-arginine’s hydrolysis to citrulline and ammonia and inhibits the arginine-dependent synthesis of NO (Figure 6). Nitric oxide is one of the main regulators of gut mucosal vasodilation [19,20]. Increased NO levels have been associated with the inhibition of hypoxia-inducible factors (HIFs) through a previously determined oxygen-consuming mechanism [21]. The intestinal dysbiosis reported in COVID-19 infected patients might dysregulate NO’s production at the intestinal epithelium level [22]. It is known that NO overproduction leads to animal morbidity and death in experimental models of influenza virus infection characterized by a state of pulmonary hyperinflammation [23].

In this context, we speculate that the administration of SLAB51 could produce a relevant inhibition of the NOS2 activity that, by limiting the blood afflux and the oxygen usage in the gut, increases the amount of O_2_ available in the arterial blood for the brain, kidney and heart. Notably, in patients with hepatitis C virus (HCV) infection with hepatocellular carcinoma, pegylated arginine deiminase (ADI-PEG 20) treatment induced NO reduction [24]. Although the inhibition of NOS2 activity in the CaCo2 cell model represents a first piece of important evidence about the molecular mechanisms supporting a reduced O_2_ consumption in the intestine, further in vitro analyses on additional cell lines are needed to confirm our result. In short, we hypothesize that SLAB51 activates an “oxygen sparing effect” detectable by the blood gas analysis and making it clinically relevant. We did not evaluate the number of bacteria reaching the different gut districts after SLAB51 administration or the fecal flora changes. However, we believe that the “oxygen sparing effect” of SLAB51 starts from the upper intestine where only a limited amount of microorganisms are present, making it possible for SLAB51 to act without the interference and competition of the massive bacterial populations present in the colon [25]. Besides, ingested foods do not reach the colon until 8–10 h later, and we registered the SLAB51 in some of our patients before this.

## 5. Conclusions

A consistent number of SARS-CoV-2 infected subjects need hospitalization for lesions affecting more than 50% of the lungs. In COVID-19 patients, as the alveoli swelling worsens, it becomes more difficult for the body to absorb oxygen. Depending on various co-factors such as age, lifestyle, diabetes, cardiovascular diseases, etc., the reduced gas exchange interface is accompanied by progressive damage to the body’s organs, in some cases, facilitating the progression of the conditions up to death. SLAB51 contains ADI, which is useful in contrasting the viral infection and mitigating the COVID-19-associated inflammatory status [4,5,6]. While waiting for the lung to recuperate its functions, the “oxygen sparing effect” of SLAB51 might be useful in the time-sensitive race to maintain oxygenation at sufficient levels and reduce the need for intensive care admissions. Additional trials must reconfirm our results, and at the same time we underline that the reproducibility of the results obtained is related to the experimental conditions described and is not extensible “a priori” to probiotics other than the one used in this study [26].

## Figures and Tables

**Table 1:** Population stratified in two groups according to the therapy: RUT or RUT plus oral bacteriotherapy. IQR: interquartile range (25°–75°).

Parameter	RUT (No. = 29)		RUT + OB (No. = 40)		*p-*Value
	Median (IQR)	No. (%)	Median (IQR)	No. (%)	
Age (years)	70 (60–77)		61 (51–74·3)		0.09
Sex (Male)		25 (86·2)		22 (55)	0.01
BMI–kg/m^3^	20 (20–22)		20 (18·8–22)		0.47
Alanine aminotransferase (ALT)–IU/L	25 (18–40)		30 (23·5–45)		0.17
Aspartate aminotransferase (AST)–IU/L	21 (19–38)		26 (18–36.3)		0.95
CT score	16.5 (15–18)		16.5 (15–18) 5		0.91
Charlson index	3 (1–4)		1 (1–5)		0.24
Drug therapy					
Antiviral (Remdesivir)		10 (34.4)		8 (20)	0.28
Antibiotic		25 (86.2)		39 (97.5)	0.19

**Table 2:** Blood parameters obtained by routine laboratory analysis at 6 h from the start of treatment. Representative patients belonging to both groups were reported.

		pO_2_/FiO_2_		pO_2_ (mmHg)		FiO_2_ (%)		O_2_Hb (%)		SaO_2_ (%)	
		0 h	6 h	0 h	6 h	0 h	6 h	0 h	6 h	0 h	6 h
RUT	Patient 1	319	278	96	63	21	28	96.6	92	98	93
	Patient 2	310	175	65	35	21	40	93	94.2	98.8	98.2
RUT+OB	Patient 3	352	390	76	86	21	21	95.3	96.9	96.9	98.9
	Patient 4	328	354	69	89	21	21	82	96	96	98

## Data Availability

Data about investigated patients and analytic methods will not be made publicly available.
